# New species and records of *Cactopinus* Schwarz with a key to species (Coleoptera, Curculionidae, Scolytinae)

**DOI:** 10.3897/zookeys.56.515

**Published:** 2010-09-17

**Authors:** Thomas H. Atkinson

**Affiliations:** 5005 Red Bluff Road, Austin, TX 78702

**Keywords:** Mexico, southwest U.S., host plants, epistomal horns

## Abstract

Three new species in the genus Cactopinus Schwarz are described from Mexico and the U.S., bringing the total of known species to 21. New host and distribution records and a new key to species are included.

## Introduction

The genus Cactopinus was described by Schwarz (1899) as a monotypic genus for Cactopinus hubbardi. Since that time an additional 17 species have been described by Blackman (1938: *pini*,*koebeli*,*rhois*), Wood (1957: Cactopinus  cactophthorus, Cactopinus spinatus; 1967: Cactopinus mexicanus; 1969: Cactopinus carinatus, Cactopinus granulifer, Cactopinus microcornis, Cactopinus nasutus, Cactopinus niger; 1983: Cactopinus atkinsoni, Cactopinus burjosi, Cactopinus granulatus, Cactopinus setosus) and Bright (1967: Cactopinus depressus, Cactopinus desertus). The genus was most recently revised by Wood (1982) whose key included only 14 species (Wood 1982). Four species were described subsequently (Wood 1983). An additional 3 species are described here, bringing the total to 21 species. All species are found in Mexico and the southwestern U.S. from Oaxaca northwards to southern California and Arizona in deserts, semi-arid scrublands, and seasonally dry forests.

The most noteworthy character of the genus is the presence of paired horns on the male epistomal process. In all species the 2 horns are apparently distinct from their base to their apex. In some species the horns are short and widely separated (Figs 2, 5). Otherwise the horns are fused at their bases and for most of their length, even though there is a clear suture between them (Figs 29, 33). In some species horns extend from the epistoma to the middle of the pronotum. In these species the horns apparently maintain some flexibility, based on the variation in position in preserved specimens. In some specimens the horn is straight (Fig. 23) while in others it is curved backwards over the frons and pronotum (Fig. 27). While the horns are not segmented, they have setae set into deep punctures along their length which might allow some flexibility. In some species the punctures are so pronounced that the horns have a serrate appearance (Fig. 33). The apex of the horns is parallel in some species (Fig. 12) or strongly divergent in others (Fig. 15) and may appear digitate (Figs 24, 28, 33). In addition to marked differences in horn length among species, the length of horns is strongly variable within species as well. In females of some species, indistinct, raised calluses are present in positions similar to the location of the base of the horns in males (Fig. 30). In addition to the primary epistomal horns, usually located in the center of the epistomal process, a short tubercle or projection is found on the anterolateral margins of epistoma near the base of the antennal insertion in most species (Fig. 2, 8, 17, 24). In Cactopinus spinatus Wood (Fig. 35) there is a distinct spine between the primary horns and this outer spine or projection.

**Figures 1–4.**
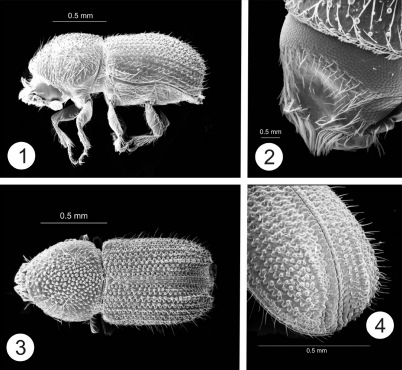
Cactopinus woodi, sp. n. **1** Male, lateral habitus **2** Male, frons **3** Male, dorsal habitus **4** Male, declivity.

Along with the development of the epistomal horns there has been a parallel anteroventral elongation of the head, particularly in males. This is evident even in species with relatively short horns (Figs 1, 10). The compound eyes have also been moved forwards and ventrally and are foreshortened by comparison with other scolytines. The male frons is generally concave from vertex to base of horns in lateral view. In some species, despite this longitudinal curvature, the resulting surface between the eyes is essentially flat (e.g., Cactopinus  cactophthorus Wood, Fig. 11, 12). In most species, however, the frons is longitudinally concave as well with a well defined fossa that varies in extent (Figs 8, 17, 19). In most females the frons is flat or convex, often transversely impressed immediately above the epistoma. The size and shape of the antennal club varies among species in size relative to the size of the head and shape (rounded or oval). Antennal sutures vary from mostly straight to procurved, or weakly bisinuate.

In most groups of the Scolytinae (*sensu* Wood 1982) asperities are found between the anterior margin and the center of the pronotum, generally with a distinct summit near the middle. In Cactopinus, this summit is invariably on the posterior margin of the pronotum and in some cases is developed into a cone that strongly projects backwards. In some species the pronotal asperities are clearly arranged in a triangular pattern with the asperities broadly distributed near the anterior margin, tapering sharply to the summit (Figs 23, 25, 27, 29). In these species there is a sharp demarcation between the asperate areas and the non-asperate posterolateral area. In most species, asperities are also found on the posterolateral portions of the pronotum (Figs 3, 6, 13).

Many specimens in collections are covered with a crust of plant resins and boring dust. This can be cleaned off by soaking in acetone or ethyl acetate overnight, combined with gentle ultrasonic cleaning. Once cleaned the elytral surface of all species is shining. At the same time, the interstrial surface of the disc is generally irregular. Strial punctures (and sometimes interstrial punctures) are usually deep, although the striae themselves are generally not impressed. Granules are frequently associated with interstriae on the declivity (Figs 4, 7, 9), to the base of elytra in some cases (Fig. 3). In some species granules are associated with striae as well. The declivity of all species is steep in lateral view, ranging from nearly convex to deeply sulcate. Lateral elevations (usually interstriae 2–4) sometimes do not extend to the costal margin and extend beyond the apex of elytra in lateral view (Figs 1, 7), appearing as lobes. The apex of the declivity is normally truncate in dorsal view but weakly acuminate in some cases (Figs 26, 39).

While each species is host specific, generally at the genus level, collectively they are found in a variety of totally unrelated hosts. Hosts include pines (2 species), Bursera spp. (Burseraceae, 2 species), Rhus spp. (Anacardiaceae, 1 species), leaves of the Agavaceae (Yucca spp., 1 species; Agave spp., 1 species), and various genera and species of columnar cacti (14 species). Actual hosts of at least half of the species breeding in cacti are not known. The only thing that the different hosts have in common appears to be co-occurrence in arid plant communities. All species have the ability to breed in apparently completely dry host material. Partly as a consequence, it is typical to see signs of breeding by multiple generations within the same piece of host material. This appears to be an adaptation to the xeric habitats where these hosts occur. While this is unusual with the Scolytinae, similar behavior is found in other groups, notably the Micracina. Based on a subjective analysis of morphological characteristics, it would appear that columnar cacti are probably the ancestral hosts and that other hosts associations are derived (widely separated vs. basally fused epistomal horns; shorter vs. longer horns; weakly developed pronotal asperities and poorly developed summit vs. strongly pronounced asperities with posteriorly projecting summit).

All species, so far as is known, are monogynous, with galleries constructed by a single pair. Which sex initiates galleries is unknown. In cases where multiple generations breed in the same piece of host material it is not known whether beetles emerge upon maturity and re-enter host tissues or whether new galleries are initiated without emerging. Galleries of several species have been described by Wood (1957) and Bright (1967) as an irregular, elongate gallery, several times wider than the width of adults, frequently filled with frass, and with multiple larval mines proceeding from poorly defined egg niches. I have seen similar galleries in Cactopinus agavensis and Cactopinus depressus. In most cactus-breeding species it is difficult to interpret adult galleries. In several of those species I have observed that galleries are initiated in the areoles (clusters of spines) along the outer ridge of the ribs. Galleries are then excavated under the tough epidermis and eggs appear to be deposited in individual niches.

The following abbreviations are used for museums where specimens are deposited: USNM(U.S. National Museum), TAMU(Texas A&M University), FSCA(Florida State Collection of Arthropods), CAS(California Academy of Sciences), CEAM(Centro de Entomología y Acaralogía, Colegio de Postgraduados, Montecillo, México).

## Systematics

### Cactopinus woodi

Atkinson sp. n.

urn:lsid:zoobank.org:act:0911530D-453A-4628-9AC8-380095EB422B

Figs 1–4 

#### Description.

This species is named in honor of Steve Wood, especially appropriate considering the large number of species in this genus that he described. It is easily recognized by its widely separated, short epistomal horns and by the large, uniform granules associated with all interstrial and strial punctures on the disc to its base and on the declivity, except for striae and interstriae 1 and 2.

##### Male.

Color black. Length 1.4–1.6 mm, width 0.6–0.7 mm, length/width 2.3. Epistomal horns short, length 2–3× basal diameter, widely separated by distance greater than length; without any associated setae. Frons weakly concave from epistoma to upper level of eyes; concavity without raised margin dorsally or laterally; surface of concavity smooth, impunctate; setae sparse, short, most abundant on periphery of concavity. Antenna subcircular, sutures slightly procurved. Pronotum with asperities concentrated near middle; summit at posterior margin, not strongly pronounced; some asperities on postero-lateral areas; area of greatest concentration of asperities not sharply demarcated laterally. Striae deeply punctured; all punctures associated with rounded granules to base of elytra; granules occupying entire space between adjacent punctures. Interstriae 1.5× as wide as striae; setae uniseriate, each associated with a rounded granule to base. Granules on striae and interstriae similar in size. Declivity steep, rounded posteriorly, strongly sulcate. Striae 1 and 2 impressed; granules absent; punctures smaller than on disc; granules also absent from associated interstriae. Interstriae 3 narrowly elevated, forming a distinct crest; its granules larger than on disc. All other declivital striae and interstriae similar to those of disc. Lateral elevations highest in middle, projecting beyond apex of elytra in lateral view.

##### Female.

Frons flattened, surface sparsely punctured, setose in central area. Other characters identical to those of males.

#### Materials examined.

**HOLOTYPE** (male): “MEXICO: Baja California, Isla San Esteban, 6-V-1985, THA-289, Stenocereus gummosus, T.H. Atkinson // Holotype Cactopinus woodi, T.H. Atkinson 2009”. **ALLOTYPE** (female): same data as holotype. The holotype and allotype are deposited in the U.S. National Museum. **PARATYPES**: Same data as holotype and allotype (60) distributed as follows: USNM-10; TAMU-16; FSCA-10; CAS-10; CEAM-10; MEXICO: Baja California, Isla San Lorenzo, 6-V-1985, THA-285, Stenocereus gummosus, T.H. Atkinson (TAMU-9); MEXICO: Sonora, Isla Tiburón, 14-V-1985, THA-301, Stenocereus thurberi, T.H. Atkinson (TAMU-2); U.S.: Arizona, Pima Co., 1 mi N Organ Pipe Cactus Natl. Mon., Hwy 85, 13-II-2008, 32.204 N, 112.754 W, Stenocereus thurberi, T.H. Atkinson (TAMU-13).

#### Notes.

This species has been collected in the dried ribs of its host cacti. As is the case with most other cactus-breeding species, successful breeding occurs in portions of stems that have dried out to a hard, yellowish color, without the black discoloration associated with decay. This situation most commonly occurs in erect, dead stems still attached to the host. In pieces that fall to the ground the upper surface is apparently too hot from direct exposure to the sun and the lower surface seems likely to decay from contact with the soil. Galleries are initiated at the areoles, clumps of spines that are found along the ridges of the ribs.

### Cactopinus sulcifrons

Atkinson sp. n.

urn:lsid:zoobank.org:act:67743115-3AE5-48DA-AF08-1EEEAD3BF89B

Figs 5–7 

#### Description.

This species is readily distinguished from other species with short, separated epistomal horns by the nearly flat male frons with only a narrow, longitudinal sulcus. This frontal sulcus is the basis for the specific epithet.

##### Male.

Color black. Length 1.4–1.7 mm, width 0.6–0.7 mm; length / width 2.4. Epistomal horns short, pointed, height slightly greater than basal diameter; located near center of epistoma, separated by 1.5× height. Frons flattened with narrow longitudinal sulcus in center, not wider than the distance between the horns. Surface sparsely punctured, with short setae; setae and punctures more abundant on periphery. Asperities on anterior margin tooth like, widely separated, becoming more abundant, flatter, and densely packed towards center and summit. Asperities are tightly packed, and slightly overlapping in a triangular pattern in the center and posterior of the pronotum, but less densely spaced asperities are abundant in posterolateral areas to the posterior margin. Striae not impressed, punctures deep, spaced within row by distance equal to their own diameters. Interstriae 1.5× as wide as striae with shallow uniseriate punctures. Vestiture of short strial setae and longer, erect interstrial setae, becoming longer posteriorly. Declivity steep, sulcate, with lateral elevations strongly elevated in middle, posterior margin rounded. All interstriae except 1–2 with rounded, uniseriate granules beginning at base of declivity. Punctures on striae 1 and 2 smaller than on disc. Lateral elevations very wide, consisting of elevated portions of interstriae 3–5.

##### Female.

Frons transversely impressed above epistoma, convex above, surface sparsely punctured, setose in central area. Epistoma with low calluses in same position as male horns. Other characters identical to those of males.

**Figures 5–10.**
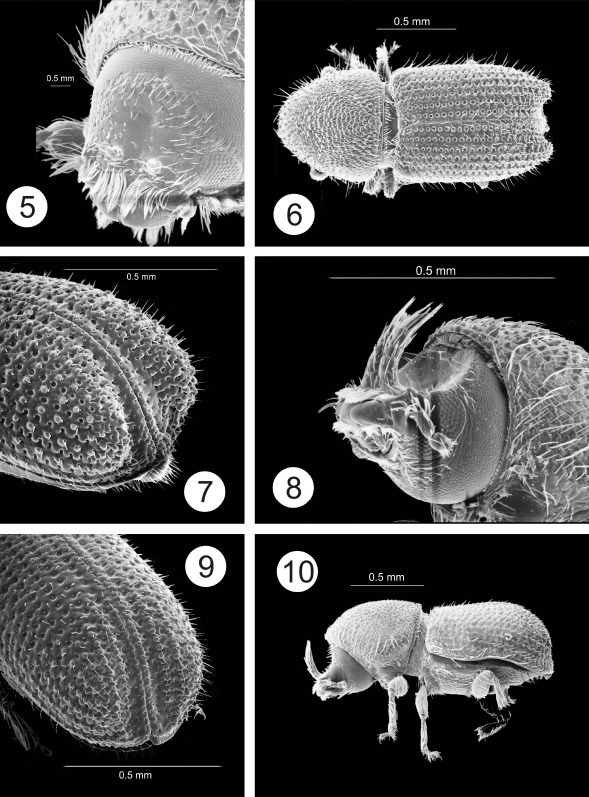
5–7 Cactopinus sulcifrons, sp. n. **5** Male, frons **6** Male, dorsal habitus **7** Male, declivity. **8–10** Cactopinus atkinsoni Wood. **8** male frons **9** Male, declivity **10** Male, lateral habitus.

**Figures 11–16.**
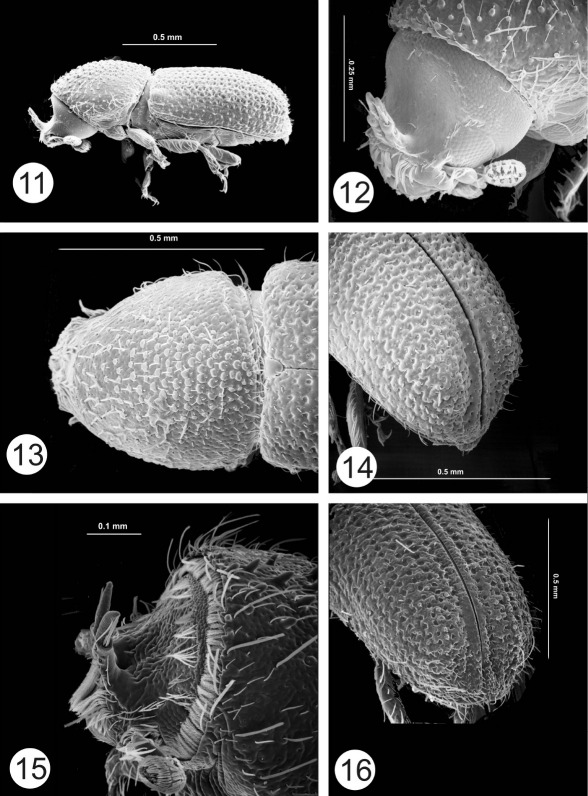
**11–14** Cactopinus cactophthorus Wood. **11** Male, lateral habitus **12** Male, frons **13** Female, pronotum **14** Male, declivity. **15–16** Cactopinus burjosi Wood **15** Male, frons **16** Male, declivity.

**Figures 17–22.**
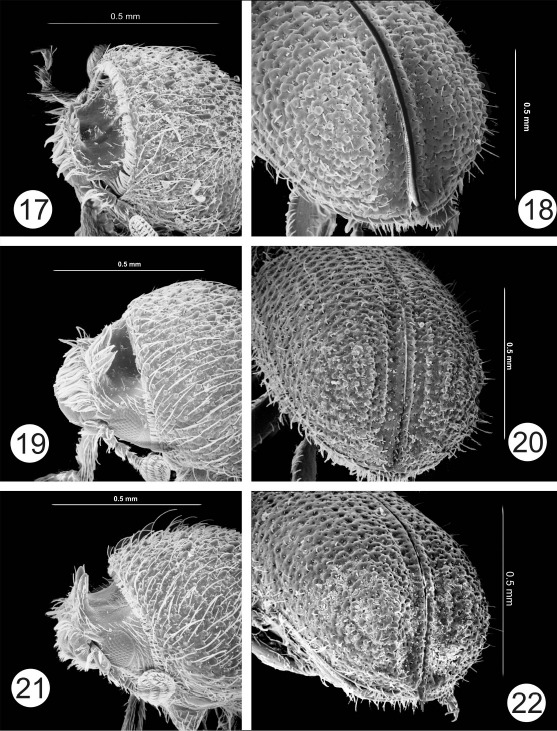
**17–18** Cactopinus carinatus Wood. **17** Male, frons **18** Male, declivity. **19–20** Cactopinus niger Wood. **19** Male, frons **20** Male, declivity. **21–22** Cactopinus setosus Wood. **21** Male, frons **22** Male, declivity.

#### Materials examined.

**HOLOTYPE** (male): “ MEXICO: Sonora, Isla Tiburón, 14-V-1985, THA-301, Stenocereus thurberi, T.H. Atkinson // Holotype Cactopinus sulcifrons, T.H. Atkinson 2009”. **ALLOTYPE** (female): same data as holotype. The holotype and allotype are deposited in the U.S. National Museum. **PARATYPES**: Same data as holotype and allotype (31) distributed as follows: USNM-2, TAMU-7, FSCA-6, CAS-6, CEAM-6.

#### Notes.

The habits of Cactopinus sulcifrons are similar to those of Cactopinus woodi.

### Cactopinus agavensis

Atkinson sp. n.

urn:lsid:zoobank.org:act:88EEB05F-250D-455C-8CEC-E160CC11404B

Figs 23–26 

#### Description.

This species most resembles Cactopinus hubbardi. It can be distinguished by the more sulcate elytral declivity with larger marginal teeth, the longer epistomal horns, and the more flattened male frons. The specific epithet is based on the name of the hosts in the genus Agave.

##### Male.

Color black. Length 1.8–2.1 mm, width 0.7–0.9 mm, length / width 2.45. Epistomal horns long, projecting to middle of prothorax, fused along their length except for the terminal ¼. Frons horizontally concave, flattened laterally with small concave area in center; surface sparsely punctured, mostly without setae. Anterolateral margins of the epistoma with short, downward projecting spine near base of antennal insertion. Antenna elongate, 1.7 times longer than wide, sutures straight. Pronotum with asperities widely separated at anterior margin, most abundant in center; arranged in sharply defined triangular pattern with no asperities or granules in posterolateral portions. Clearly defined summit at posterior margin, strongly elevated into a point, projecting backwards over elytra. Striae not impressed, with deep punctures, separated by less than their own diameter. Interstriae not elevated, about twice as wide as striae; surface irregular, with numerous, fine, confused punctures. Vestiture of recumbent, short, strial setae, with long, ribbon-like interstrial setae, these longer than distance between rows. Declivity weakly sulcate, gradual, slightly acuminate posteriorly. Interstriae 1 and 2 wider on declivity than on disc. Punctures on striae 1 absent beyond declivital base, interstriae 1 and 2 with numerous, small, confused punctures. Interstrial granules on all other declivital interstriae except 1. Interstriae 2 strongly elevated, its granules slightly longer and sharper than those on other interstriae.

##### Female.

2 wide calluses present on epistoma, frons transversely impressed above. Frons shallowly concave above transverse impression, surface with shallow, large, closely set punctures. A fringe of setae along upper and lateral margins of convavity. Other characters identical to those of males.

**Figures 23–26.**
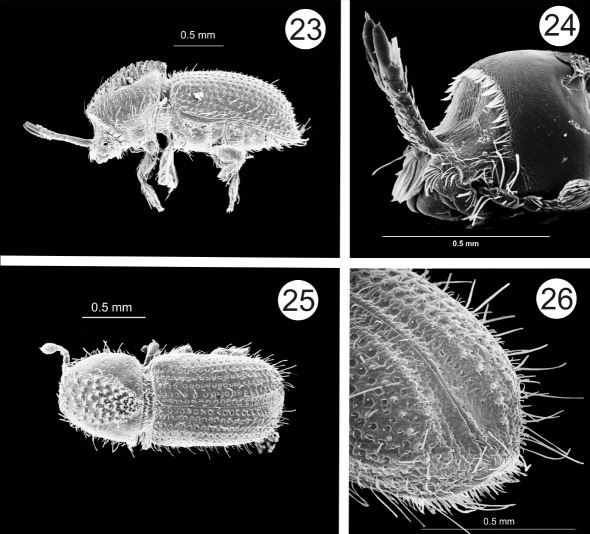
Cactopinus agavensis, sp. n. **23** Male, lateral habitus **24** Male, head **25** Female, dorsal habitus **26** Male, declivity.

#### Materials examined.

**HOLOTYPE** (male): “Mexico: Mexico (state), Teotitlán, 2-VI-1982, 2,410 m, leaves Agave atrovirens, A. Equihua M. // Holotype Cactopinus agavensis, T.H. Atkinson 2009”. **ALLOTYPE** (female): same data as holotype. The holotype and allotype are deposited in the U.S. National Museum. **PARATYPES**: Same data as holotype and allotype (11) distributed as follows: USNM-2; TAMU-5; CEAM-4. MEXICO: Mexico (state), Noplatepec, 11-VII-2008, leaves Agave salmiana, T.H. Atkinson & A. Equihua M. (TAMU-2, FSCA-5, CAS-5, CEAM-4); MEXICO: Guanajuato, Hwy 57, 9.6 km S intersection Hwy 110, 21.19881 N, 100.57501 W, 6-VII-2009, 1,989 m, leaves Agave salmiana, T.H. Atkinson, THA 902 (TAMU-7, CEAM-6).

#### Notes.

This species has been collected on 3 occasions in the central Mexican highlands from the large semi-domesticated agaves used historically for pulque production. These plants reach a very large size with individual leaves reaching a length of 1.5 m or more and armed with strong, recurved spines. The insect is found in dead, mostly dry leaves at the bottom of the rosette. In a healthy plant, the only way to get to these leaves is to basically take the plant apart, something that requires a lot of work and potential loss of blood on the part of would-be collectors. It is more easily collected from rosettes that are dying and beginning to fall apart after blooming, or in the occasional specimen growing on the edge of a terrace or wall such that the lower leaves can be reached. The beetles may enter the leaves from either the top or bottom surface and galleries resemble those described by Wood (1982). Apparently multiple generations may develop in the same dried leaf until it is consumed.

## Key to males of Cactopinus

Information on hosts and distribution is included in the key as a general aid to users. Distributional information should only be used in a very general sense given that many species are still known only from type localities.

**Table:** 

1	Asperities on pronotum large, chisel-like, forming distinct triangle with apex at posterior margin, clear demarcation between this area and posterolateral portions of pronotum, most asperities posterior to middle (Figs 23, 25, 27, 29); antennal club narrow, 1.7–2 times as long as wide, sutures straight (Fig. 33); hosts not restricted to cacti	14
–	Asperities on pronotum small, often granulate, more evenly distributed, not in clearly marked triangular pattern, apex indistinct or not strongly projecting backwards; most asperities anterior to middle (Figs 3, 6, 13); antennal club rounded, less than 1.3 times as long as wide, sutures weakly procurved to bisinuate (Fig. 21); all hosts columnar or arborescent cacti	2
2(1)	Male epistomal horns clearly separated at base (Figs 2, 5)	3
–	Male epistomal horns contiguous at base and fused along lower ? or more of length (Figs 8, 12)	6
3(2)	Epistomal horns 4–5 times longer than basal diameter, separated by less than half their length; frons deeply concave, abruptly margined at top. 1.3–1.6 mm. Jalisco	Cactopinus mexicanus Wood
–	Epistomal horns less than twice basal diameter (Figs 2, 5); frons weakly excavated, margins not abrupt	4
4(3)	Epistomal horns about twice as long as basal diameter, separated by more than twice their length; frons shallowly concave; prominent granules on all declivital striae and interstriae to base. 1.4–1.6 mm. In Stenocereus spp. Arizona, Baja California, Sonora (Figs 1–4)	Cactopinus woodi Atkinson
–	Epistomal horns about as long as basal diameter, separated by slightly more than height; granules restricted to posterior portion of elytra and declivity	5
5(4)	Male frons concave over entire area between eyes, concavity extending beyond upper level of eyes; lateral convexities on declivity weakly elevated. 1.5–1.9 mm. Oaxaca	Cactopinus microcornis Wood
–	Male frons flattened, with narrow, shallow longitudinal impression from epistoma to upper level of eyes; lateral convexities on declivity abruptly, strongly elevated. 1.4–1.7 mm. In Stenocereus spp. Sonora. (Figs 5–7)	Cactopinus sulcifrons Atkinson
6(2)	Granules present at least on posterior portion of discal interstriae as well as on declivity	7
–	Granules on elytral interstriae and /or striae restricted to declivity	8
7(6)	Frons deeply concave, concavity occupying entire distance between eyes, wider above eyes; declivity moderately sulcate, granules present on all interstriae. 1.6–1.8 mm. Jalisco	Cactopinus granulatus Wood
–	Frons less deeply concave, concavity occupying 80% of distance between eyes, not wider above eyes; declivity deeply sulcate, interstriae 1 and 2 shining, without granules. 1.3–1.6 mm. Oaxaca	Cactopinus granulifer Wood
8(6)	Frons deeply concave, concavity occupying 90–100% of distance between eyes	9
–	Frons shallowly concave or flattened, concavity if present occupying 60% or less of distance between eyes	11
9(8)	Upper part of concavity of frons wider above eyes, upper margin acute; declivity more pronounced, interstriae 2 narrowed, without granules except near base	10
–	Upper part of frontal concavity not wider above eyes, upper margin less pronounced; declivity shallower, interstriae 2 not narrowed, with granules for its full length. 1.3–1.5 mm. In Stenocereus spp. Jalisco. (Figs 8–10)	Cactopinus atkinsoni Wood
10(9)	Upper half of frons bearing longitudinal carina; apex of pronotal summit not developed into backwards-projecting cone. 1.5–1,8 mm. In Myrtillocactus. Hidalgo, San Luis Potosi, Tamaulipas. (Figs 17, 18)	Cactopinus carinatus Wood
–	Upper half of frons without longitudinal carina; apex of pronotal summit developed into backwards-projecting cone. 1.4–1.7 mm. Puebla, Oaxaca	Cactopinus nasutus Wood
11(8)	Frons curved in lateral profile, but flat longitudinally; pronotal asperities weakly developed; declivity with relatively few, small granules on interstriae. 1.2–1.4 mm. Puebla. (Figs 29–32)	cactophthorus Wood
–	Frons weakly to prominently concave in central area between eyes; pronotal asperities larger; declivity with prominent granules on interstriae 2 and higher	12
12(11)	Horns reaching or exceeding upper level of frons, apical portions digitate, divaricate; interstria 1 on declivity not deeply impressed, lateral elevations not pronounced. 1.5–1.6 mm. In Neobuxbaumia. Morelos, Puebla. (Figs 11–14)	Cactopinus burjosi Wood
–	Horns not reaching upper level of frons, outer sides parallel to apex, interstria 1 on declivity strongly impressed, lateral elevations prominent	13
13(12)	Frons with prominent concavity in middle of frons; outer sides of horns parallel, inner sides angled making obvious “V” shape; antennal sutures straight. 1.6–1.9 mm. In Stenocereus. Oaxaca, Queretaro. (Figs 19, 20)	Cactopinus niger Wood
–	Frons shallowly concave, concavity not well defined; inner sides of horns not strongly angled; antennal sutures bisinuate. 1.4–1.7 mm. In Stenocereus spp. Jalisco. (Figs 21, 22)	Cactopinus setosus Wood
14(1)	Declivity strongly sulcate, lateral elevations armed with teeth larger than granules on other interstriae, sutural interstriae depressed, widened prominently in middle; interstriae 2 curved outwards in compensation	15
–	Declivity weakly or not sulcate, sutural interstriae not widened on declivity, interstriae 2 not curved outwards	19
15(14)	Declivity with obvious teeth on interstriae 2 on lateral margin of declivity much larger than granules present on other interstriae	16
–	Declivity with granules on interstriae 2 only slightly larger than those on other interstriae	17
16(15)	Horns 1.5–2 times as long as frons; apex of pronotal asperities strongly pronounced, backwards projecting; teeth on interstriae 2 flattened laterally, height greater than twice basal width. 1.6–2.1 mm. In Bursera spp. SW U.S., NW Mexico. (Figs 32–34)	Cactopinus desertus Bright
–	Horns shorter, slightly longer than length of frons; apex of pronotal asperities not strongly pronounced; teeth on declivital interstriae 2 cone shaped, about 1.5 times as high as basal width. 1.4–1.7 mm. In Bursera spp. Oaxaca, Morelos, Jalisco. (Figs 35, 36)	Cactopinus spinatus Wood
17(15)	Body slender, 2.6 times longer than wide; interstriae 1 strongly impressed, sulcate anterior to declivity. 1.6–2.0 mm. In phloem of of Pinus spp. SW U.S.	Cactopinus pini Blackman
–	Body stouter, 2.4 times longer than wide; interstriae not impressed or sulcate anterior to declivity	18
18(17)	Frons weakly concave, impunctate; interstriae 1 and 2 on declivity not widened, granules on interstriae 2 small, rounded, restricted to base. 1.6–2.3 mm. In Carnegiea gigantea. Arizona, probably Sonora. (Figs 27–31)	Cactopinus hubbardiSchwarz
–	Frons weakly flattened longitudinally, densely, finely punctured; interstriae 1 and 2 on declivity widened in middle, granules on interstriae 2 larger than those on other interstriae, pointed, present to apex. 1.8–2.1 mm. In dry fleshy leaves of Agave spp. State of Mexico, Guanajuato. (Figs 23–26)	Cactopinus agavensis Atkinson
19(14)	Declivity with coarse granules on interstriae 1–3. 1.3–1.8 mm. In Rhus spp	SW U.S. Cactopinus rhois Blackman
–	Declivity without granules on interstriae 1–3	20
20(19)	Horns short, not reaching top of frons; elytral punctures shallow; granules not present on declivity, declivity narrowly sulcate. 1.3–1.5 mm. In leaves of Yucca spp. San Luis Potosi, Hidalgo, Nuevo Leon. (Figs 37–40)	Cactopinus depressus Bright
–	Horns longer, exceeding top of frons; elytral punctures deeper; declivity not sulcate. 1.3–1.5 mm. In phloem of of Pinus spp. SW U.S., Baja California	Cactopinus koebeli Blackman

## New host and distribution records

New collection records are only shown if they represent significant geographic range extensions or new host associations.

**Figures 27–32.**
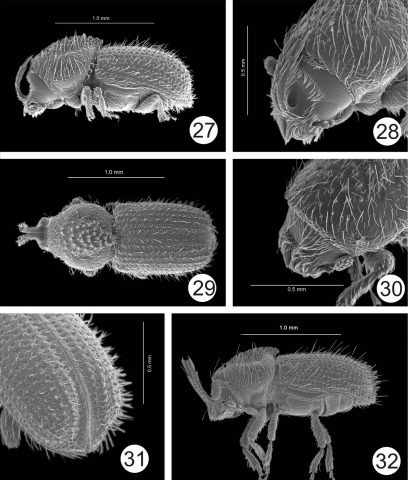
**27–31** Cactopinus hubbardi Schwarz. **27** Male, lateral habitus **28** Male, frons **29** Male, lateral habitus **30** Female, frons **31** Male, declivity. **32** Cactopinus desertus Bright, male, lateral habitus.

### Cactopinus carinatus Wood

Previously known only from the type locality in Hidalgo from “giant cactus”. Mexico: San Luís Potosí: Hwy 57, 18 km S intersection Hwy 80; 22.76227 N, 100.49222 W; 6-VII-2009; 1,422 m; branches of Myrtillocactus geometrizans, coll. T.H. Atkinson (TAMU-8, CEAM-6); Mexico: Tamaulipas: outside Tula; 23.01077 N, 99.73729 W; 16-VIII-2009; 1,164 m; branches Myrtillocactus geometrizans, T.H. Atkinson (TAMU-4, CEAM-6, FSCA-4). These collections represent new state records and the first known host records for this species. Myrtillocactus geometrizans is one of the most widespread of the arborescent-columnar cacti in Mexico and is easily recognizable. It is known to occur in the vicinity of the type locality for Cactopinus carinatus. While it is premature to conclude that this species is the only host for Cactopinus carinatus, it is significant that it has not been collected in numerous recent collections from other species of cacti.

### Cactopinus niger Wood

Previously known only from the type locality in Oaxaca from “giant cactus”. Mexico: Querétaro: Hwy 57D, 15 km N intersection Hwy 45D; 20.67390 N, 100.3046 W; 2,048 m; 6-VII-2009; dry stems of Stenocereus queretaroensis; T.H. Atkinson (TAMU-4, CEAM-4). This is the first known host record.

### Cactopinus desertus Bright

Reported previously from southern California in the U.S. and from the Mexican states of Baja California and Sonora. Mexico: Baja California Sur; San José del Cabo, 11 mi SW; 28-VI-1967; blacklight; E.L. Sleeper, E.M. Fisher (CAS-1); BCS: Santa Victoria, 27/28-X-1968; 800’; blacklight; E.L. Sleeper, F.J. Moore (CAS-1); BCS: Loreto, 3 mi N; 10/11-XI-1968; blacklight; E.L. Sleeper, F.J. Moore (CAS-1)

**Figures 33–36.**
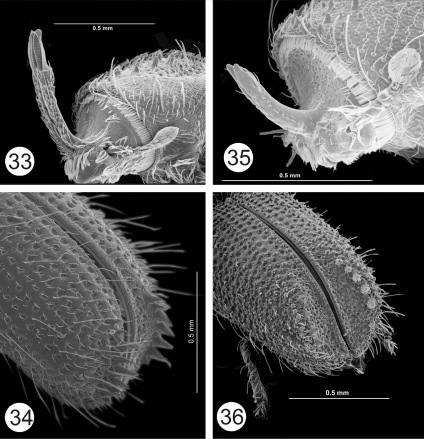
**33–34** Cactopinus desertus Bright. **33** Male, frons **34** Male, declivity. **35–36** Cactopinus spinatus Wood **35** Male, frons **36** Male, declivity.

### Cactopinus spinatus Wood

Previously the only known hosts were tropical trees and shrubs of the genus Bursera, although Wood (1982) indicated that he had collected it from hosts that did not appear to be of that genus. Mexico: Oaxaca: Cuicatlán, 5 km N; 17.74010N, 96.94901 W; 665 m; 2-VII-2009; Cyrtocarpa procera (Anacardiaceae), T.H. Atkinson (TAMU-16, FSCA-8, CAS-8, CEAM-8). The families Burseraceae and Anacardiaceae are closely related both taxonomically and chemically.

### Cactopinus rhois Blackman

This species has previously been reported from Los Angeles and Ventura Counties in southern California. CA: San Diego Co., Oak Grove Campground, Cleveland N.F.; 3-XII-1991; Rhus trilobatus; T.H. Atkinson, D.W. Hawks, L..R. Kirkendall (TAMU-2).

### Cactopinus depressus Bright

Reported previously from the states of San Luís Potosí and Hidalgo. New Records: Mexico: Nuevo León: Hwy 57, y mi S. Entronque Roberto; 20-VI-1983; 1,768 m; from dry Yucca leaves; C.W. and L. O’Brien, G.B. Marshall (FSCA-5); Nuevo León: Mier y Noriega, 12 km NW; 10-XI-1976; 1,460m; from dry Yucca leaves; A.N. García Aldrete (FSCA-1).

**Figures 37–40.**
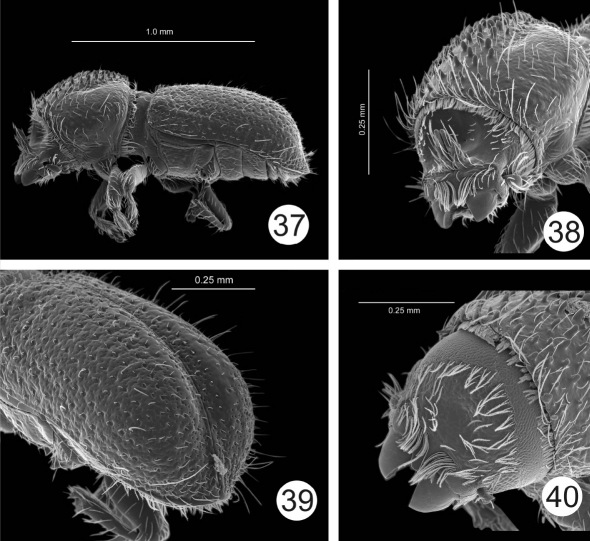
Cactopinus depressus Bright. **37** Male, lateral habitus **38** Male, frons **39** Male, declivity **40** Female, frons.

## Supplementary Material

XML Treatment for Cactopinus woodi

XML Treatment for Cactopinus sulcifrons

XML Treatment for Cactopinus agavensis
